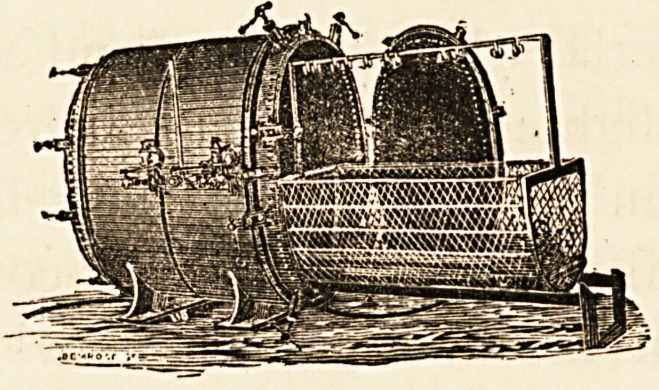# Notes on Disinfection
*For full details upon this subject consult the New Sydenham Society's vol. cxv., *Recent Papers on Disinfection;* and Dr. Parsons' *Report on Disinfection by Heat*—extracts from the *Annual Report of the Medical Officer of the Local Government Board* for 1884 (Eyre and Spottiswoode). An excellent paper by Dr. Whitelegge also appeared in the *Sanitary Record* for March, 1887.


**Published:** 1888-06

**Authors:** D. S. Davies

**Affiliations:** Medical Officer of Health, City and County and Port of Bristol


D. S. DAVIES, M.B. Lond., D.P.H. Cantab.,
*Medical Officer of Health, City and County and Port of Bristol.*

---

ONE of the chief obstacles to an accurate study of disinfection-processes has, in former years, been the difficulty of deciding upon the test by which a true disinfectant should be known, as distinct from an antiseptic or a mere deodorant.

The demonstration in recent years of the actual contagium of certain specific infectious diseases, *e.g.* anthrax and tuberculosis—the, as yet, less complete demonstration of the contagious principle in others—and the extremely well-supported inference, from analogy, that the common infectious disorders all depend for causation upon specific micro-organisms, have placed in our hands a readily available and trustworthy test of disinfectant properties.

It seems now to be universally agreed that a true disinfectant must have the power of destroying all living micro-organisms and their spores—not merely that of

* For full details upon this subject consult the New Sydenham Society's vol. cxv., *Recent Papers on Disinfection*; and Dr. Parsons' *Report on Disinfection by Heat*—extracts from the *Annual Report of the Medical Officer of the Local Government Board* for 1884 (Eyre and Spottiswoode). An excellent paper by Dr. Whitelegge also appeared in the *Sanitary Record* for March, 1887.

checking their growth. The resisting properties of the various micro-organisms to chemical and other methods of disinfection vary considerably; and the most resistant bodies of all at present known are the *resting spores* of bacilli. Spore-forms may be suspected in diseases such as smallpox, the contagia of which retain their virulence for long periods of time in the dry state. It is therefore essential that a disinfectant which is to be employed against contagia of unknown, and possibly highly resistant, properties shall be able to destroy the spores of bacilli absolutely and beyond all possibility of recuperation, as shown by the failure of subsequent artificial cultivation.

It is true, as Koch points out, that for strict accuracy, it would be necessary to test a disinfectant separately against each and every form of virus which it may be called upon to destroy, and under the same conditions as in actual practice; as, though the probability is very great, it has not yet been shown to demonstration that *all* infective material is organised; and, again, it is probable that the respective organisms may be very differently constituted, and therefore very differently affected by the same reagent.

Practical experience, however, tends to show that the contagia of the common infectious diseases are less resistant than the spore-forms used in these experiments, and that, consequently, in accepting these conclusions we not only ensure complete disinfection, but obtain, in addition, a considerable margin of safety.

The subject naturally divides itself into three heads:

1. Disinfection by heat.
2. Disinfection by chemicals in solution.
3. Disinfection by chemicals in the form of vapour.

*Disinfection by Heat (Dry Heat and Steam Heat).*

The earliest experiments on heat-disinfection, as distinguished from destruction by fire, were those made by Dr. Henry, of Manchester, *circ.* 1831. His experiments consisted partly of observations on the effect of heat upon vaccine lymph, and partly of experimental attempts to infect persons and children by causing them to wear flannel clothing exposed to certain degrees of heat (a little over 200° F. for some hours) removed from "typhus" and "scarlet fever" patients. No injurious effects appear to have followed; but it was not shown that the persons experimented upon were susceptible to the infection, nor was the proof completed by causing the same persons to wear garments equally infected, but unheated. Experiments of this kind do not appear, for obvious reasons, to have been extensively followed. Passing over many and various experiments on heat-disinfection between the years 1831 and 1881, we come at length to a time when the demonstration of the part played by micro-organisms in the causation and spread of certain specific infectious diseases, and the very well-founded inference that they may play a similar part in most or all diseases of like character, had, as has been seen above, led us to the belief that, by ascertaining the action of certain modes of disinfection upon the most resistant micro-organisms, we may know when we have secured efficient disinfection.

In 1881, Koch, Wolfhügel, Gaffky, and Loeffler made various important experiments on dry and moist heat processes of disinfection, in continuation of their experiments upon the action of chemical agents.

Koch's actual process is thus described:—"Pure cultivations were obtained of species of bacteria which are not liable to occur in accidental impurities derived from the air, and which at the same time possess striking and characteristic peculiarities. Solid cultivating material was employed in place of liquid, in order to avoid the necessity of complex precautions. Small quantities of the respective pure cultivations having been exposed to the action of disinfectants, were transferred to solid nutrient jelly or potato, and the resulting growth, if any, compared with that occurring in a control experiment which was made in every instance. By these means such complete evidence is obtained as to the vitality of the respective bacteria, both before and after disinfection, that errors are entirely excluded."

Still more recently, the experiments of Koch on heat-disinfection have been confirmed and extended in the course of an elaborate investigation by Drs. Parsons and Klein. Their experiments, made upon artificial cultures of the anthrax bacillus, upon spores of the same, upon artificial cultivations of swine fever, tuberculous pus, and upon lice, and embracing the questions of penetration into non-conducting articles, liability to injury of articles submitted to disinfection, and the degree and duration of heat necessary for disinfection, showed that—

1. The spores of anthrax bacillus were destroyed by five minutes' exposure to a heat of 212° F. in steam or boiling water; but required for destruction four hours' exposure to dry heat of 220° F., or one hour's exposure to dry heat of 245° F. The infective materials destitute of spores were completely destroyed by five minutes' exposure to steam at 212° F., or by one hour's exposure to dry heat at 220° F.

2. The penetrative power of steam into bulky or badly-conducting articles, such as bedding, is vastly superior to that of dry heat, and is much aided by employing it under pressure, and relaxing the pressure from time to time, so as to displace the cold air in the interstices of the material.

3. Scorching occurs very readily with dry heat when the temperature at all exceeds 250° F., and white wool is soonest affected; it is especially apt to occur with radiant heat, as when the disinfecting chamber is directly heated from below. Both dry heat and steam will "fix" indelibly stains in linen or blankets soiled by discharges. Steam heat causes a certain amount of shrinkage in cloth goods, about as much as an ordinary washing; but its wetting effect is diminished by surrounding the disinfecting chamber with a steam-jacket containing steam at a higher pressure than in the interior, so as to superheat the steam in the interior chamber. Steam causes no appreciable injury, except to leather, which is entirely destroyed.

4. The objects for which disinfection by dry heat or steam is particularly applicable are such as will not bear boiling in water, *e.g.* bedding, blankets, carpets, and cloth clothes generally. When things may be boiled without injury, linen for example, this is by far the readiest mode of disinfection; the grosser dirt being first removed by preliminary soaking, and precautions being of course taken against re-infection during the process.

5. With dry heat the prolonged heating to such high temperatures (above 250° F.) caused injury to most objects submitted to the process.

6. The results obtained with steam were strikingly superior. An exposure of five minutes to steam at 212° F. was sufficient to kill the spores of the anthrax bacillus, and one of 15 minutes to kill spores of the bacilli contained in garden earth.

7. The three important requirements for a satisfactory disinfecting machine are:

(*a*) That the temperature of the interior shall be equally distributed;

(*b*) That it shall be capable of being maintained constant for the time during which the operation extends; and

(*c*) That there shall be some trustworthy indication as to the actual temperature of the interior at any given moment, so that scorching may be avoided on the one hand, and inefficient disinfection on the other.

The directly-heated dry air chambers showed most unequal distribution of heat, variations of 100° F. being observed in different parts of the interior. Those heated by warm air currents were superior in this respect; and in the better gas-heated ones (as the Nottingham stove) the temperature is automatically regulated. But all forms of dry heat apparatus are very slow in securing the necessary penetration; the attached thermometer also, being often placed in a guarded spot, and thus sheltered, differed from the actual interior temperature in some cases by as much as 100° F.

8. Steam apparatus comply with each one of the above requirements.

*Chemical Disinfectants in Solution.*

While, however, the labours of these enquirers have established upon a sufficiently secure basis the efficiency of heat-disinfection for those purposes to which it is applicable, the continuation of similar experiments upon the chemical disinfectants in common use has considerably modified our views as to their value.

Thus, *carbolic acid*, 5 per cent. solution (1 in 20), took more than twenty-four hours to kill anthrax *spores* freely exposed to it, and a 3 per cent. solution took seven days; but even 1 per cent. in watery solution destroyed anthrax *bacilli*, and a little more than 1 per 1000 prevented the growth of bacilli. One part of thymol in 80,000 was found to impede the growth of anthrax spores. Carbolic oil, 5 per cent. solution, had absolutely no effect on anthrax *spores* in 110 days (upwards of three months); and even the sensitive anthrax *bacilli* were not more affected by 5 per cent. solution of carbolic acid in oil than by pure oil. The same holds good as regards thymol and salicylic acid. The alkaline carbolates and sulpho-carbolates proved to be less powerful than the pure acid, and therefore little to be relied upon as true disinfectants.

*Zinc chloride*, in 5 per cent. watery solution, had not in any way affected anthrax spores at the end of a month, and 1 per cent. failed to destroy the less resistant micrococcus prodigiosus in 48 hours, although its development was retarded thereby in 16 hours. These results are noteworthy in the case of a disinfectant which has been considered effective in solutions of 1 per 1000.

*Sulphate of iron* had little effect even in preventing ordinary putrefaction, and at the end of six days a 5 per cent. solution had not in any way affected anthrax spores.

*Chloride of lime*, in 5 per cent. solution, retarded the development of anthrax spores at the end of the first day; they were still alive after the second day, but were destroyed after five days' exposure.

*Potassic permanganate*, in 5 per cent. solution, destroyed anthrax spores in one day; but 1 per cent. solution was

8

ineffective after two days. It must be remembered, as Dr. Whitelegge points out, that the permanganate in the experiment constituted 5 per cent. of the whole body of liquid, and that if any non-living organic matter be present, there must be allowed sufficient permanganate to oxidise the whole of it, and to leave, *in addition*, the requisite 5 per cent. to act upon the infective material. These quantities would be very much in excess of those employed in ordinary practice.

*Mercuric chloride.*—Koch found that this salt, in the enormous dilution of 3 parts per million, entirely arrested the development of anthrax *bacilli*, and that 1 part in 5000 destroyed anthrax *spores* in ten minutes.

Mercuric chloride would therefore appear to be the best and most reliable disinfectant; and it is fortunately cheap, especially in the dilute solutions (say 1 per 2000) necessary for use. The chief drawbacks to its extended use are, the risk from its extremely poisonous character, which may be partly obviated by using litmus-coloured solutions; and the fact that in albuminous liquids, or in the presence of sulphides, it forms inert precipitates. Koch suggests that the mercury solution should continue to be added until bright strips of copper left in the liquid for half an hour show a distinct formation of amalgam, indicating the presence of at least 1 part per 5000 available mercuric chloride in solution.

### *Chemical Disinfectants in the form of Vapour.*

Koch's experiments show that to ensure destruction of the spores of earth or of anthrax, exposure to *carbolic vapour* for five or six hours, at a temperature of 75° C. (167° F.), would probably be necessary. It would thus be inapplicable to the disinfection of rooms; but carbolic vapour, with moist heat, promises good results in a special apparatus—results, however, which are equally well obtained by the use of moist heat alone. Similarly, the vapour of bisulphide of carbon destroyed the spores in two hours, at a temperature of 80° C. (176° F.)

*Sulphurous acid gas—Chlorine and Bromine gas.*—For details of the many important experiments upon the use of these reagents in disinfection, the reader is referred to the papers of Koch, Fischer, and Proskauer, in the Sydenham Society's volume, cxv. The chief results are thus summed up by Dr. Whitelegge: "Sulphurous acid signally failed to affect dry anthrax spores even when the proportion of gas was many times greater than it ever is in practice, though well-soaked spores were destroyed. In ordinary rooms the loss of sulphurous acid was found to be very rapid; and, in the absence of moisture, the effect upon even comparatively sensitive micro-organisms, such as micrococci, was *nil,* although the gas was in double and treble the usual proportion (1 lb. sulphur burnt for every 1000 cubic feet of air-space—giving theoretically 1.1 per cent. $SO_2$); and the observation extended over two days." But anthrax bacilli were quickly destroyed if exposed directly to the action of the gas in very thin layers, as, for instance, upon silk threads. "Finally, samples of anthrax spores were placed in crevices and in pockets of garments, in a room in which ten times the usual allowance of sulphur was burnt, the air being, moreover, saturated with moisture; after 24 hours' exposure, not one sample was sterilised. Baxter found that, with high concentration, sulphurous acid was more destructive to vaccine virus than even chlorine; but the proportions were far greater than those attainable in ordinary practice. With chlorine the results were considerably more favourable; but only when the air was saturated with moisture. It appeared that in closed vessels, where the loss of chlorine is small, 0.3 per cent. destroys all organisms in three hours ; but if the humidity falls short of saturation, not less than 1 per cent. is necessary. The experiments were continued in ordinary rooms, and samples of anthrax spores and other organisms were placed in crevices and in folds of clothing, as well as in the open, so as to imitate the conditions of actual disinfection. Almost all the organisms freely exposed had been destroyed, but very few of those sheltered from the gas by even a covering of blotting-paper, and none of those which lay in pockets or crevices, or beneath glass plates. The volume of chlorine generated in these experiments was theoretically 1.4 to 1.5 per cent. of the air-space; at the end of the first half-hour the proportion had already fallen to 0.4. Bromine was found to act very similarly to chlorine: 0.2 per cent. was sufficient for the destruction of anthrax spores in a closed vessel, the air being saturated with moisture." But this chemical presents no advantages, and is more costly to produce than chlorine.

### METHODS OF TRANSFERENCE OF CONTAGION FROM THE SICK TO THE HEALTHY.

It has commonly been taught, even by recent writers, that the contagious particles are capable of escaping with the water which evaporates from liquids or from moist solid surfaces, and of diffusing themselves in the air. Under this supposition, therefore, it has been readily accepted that the breath of a person suffering under an infectious disease is apt to be highly charged with the contagion, and that the exhalations from his moist skin, or from his excreta, may be highly dangerous. Nägeli, however, has shown, apparently to demonstration, and subsequent experimenters are confirming his results, that it is extremely difficult or impossible to set free micro-organisms from moist surfaces; and it therefore appears probable that infection, when air-borne, occurs by means of contagious particles which have undergone desiccation, and which rise into the air, and remain suspended in it, in the form of fine dust. There can be little doubt that the infectious particles of a contagion leave the body mainly in the fluid secretions and excretions—the fæces, the urine, and the sweat,—and must therefore undergo desiccation before they can reach the air. They may also be attached to the solid particles of cuticle which are constantly being shed from the surface. These are already dry, and in the most favourable condition for transmitting infection, not only directly, but also by attaching themselves to articles of clothes, bedding, or furniture, or by settling upon the walls and floors of an apartment—a point of the extremest importance, as is shown by daily experience in the case of such diseases as smallpox or scarlet fever, in which desquamation of highly infectious particles is a marked feature during convalescence.

It is sufficiently obvious that linen or clothes soaked with infective secretions are peculiarly well calculated to spread infection: the contagious particles remain upon the surface of the material when it dries, and are shaken off into the air on the slightest movement—a circumstance to which washerwomen no doubt owe their liability to infection. Although disease is undoubtedly conveyed in very many instances in the above method, we must not forget the clear evidence that the germs of certain infectious diseases—notably typhoid fever and cholera—are frequently, though not always, transmitted by means of liquids which are swallowed, and which contain the infective material. Such liquids, and milk in particular, appear also to serve as a cultivation material in which the micro-organisms multiply rapidly, in the interval between contamination and ingestion. Nor was this fact forgotten in dealing with cholera in Bristol in 1866, when absolute prohibition of possibly polluted water-supplies formed an integral part of the precautions adopted, with much success, for its suppression.*

PRACTICAL ASPECTS OF DISINFECTION.

In dealing with the subject of disinfection practically, it is obvious that we must select a different method, according as we wish to secure disinfection of infected clothing or bedding, to render harmless infectious discharges, or to purify the interior of an infected room.

To secure the first, the use of superheated steam is seen to be at once expeditious and reliable. The apparatus in use by the Bristol Sanitary Authority is one of Washington Lyon's steam disinfectors, made by Messrs. Manlove, Alliott, and Fryer, of Nottingham. Its internal capacity is large enough to contain any sized mattress, and it is surrounded by a steam-jacket, which admits of superheating, and so drying, the steam in the central chamber. Steam is supplied from a separate boiler. The machine can be got into working order in an hour, and a roomful of bedding and clothes can be disinfected in another hour. So rapid is the working of this machine,

* *Cf.* Dr. Budd's *Asiatic Cholera in Bristol in* 1866. W. C. Hemmons, Bristol. 1883.

that more than 10,000 articles have been disinfected thoroughly during the first three months of 1888—an amount of work that, with the two gas-heated dry-air disinfectors in use here previous to 1887, would have been impossible.

During the conduct of a sick case at home, as, for instance, of scarlet fever (the home-nursing of smallpox, under ordinary conditions, I hold to be inadmissible), the necessity of dealing with infected linen is continually arising. This, as has been shown, need only be boiled to ensure complete disinfection; but, while it awaits boiling, it should be steeped in a solution either of corrosive sublimate (1 in 1000), or in carbolic acid (2 per cent.), or thymol (1 in 1000), all of which, in these strengths, have at least the power of impeding the development of, and the first of destroying, anthrax spores. The infected articles should also, for considerations above stated, be kept constantly wet until actually transferred to the boiling water. No other process that can be conducted at home, except destruction by fire, can be relied upon as an efficient means of disinfection for clothes. Fumigation by sulphur or chlorine vapour is worse than inefficient, as it tends to promote a false feeling of security.

The use of chemical disinfectants in solution is especially indicated where infectious excretions or discharges are to be dealt with. In such cases it is particularly necessary that all discharges should be *at once* received into, well mixed with, and covered by, chemicals of sufficient strength. An equal bulk of mercuric chloride solution (1 in 1000) is probably the most suitable. The discharges may then be disposed of as usual. Carbolic acid, though too feeble in its action upon resistant and spore-bearing contagia, destroys bacilli in 1 per cent.

solutions, and checks their growth in even more dilute solutions—a property which may explain the excellent results obtained by the careful use of this and other accepted disinfectants.*

During the continuance of such infectious disease in a house, it is also well to keep all drains and waste-pipes flushed with chemicals; but it must clearly be remembered that on no account should such chemicals be trusted to cover or remove drain smells in a house or street. The presence of such smells points to defective construction, and the danger will remain until reconstruction on correct principles is secured.

Public sewers, again, should be constructed so as to carry off the sewage at once to a safe point of discharge, and so to prevent putrefaction; but their systematic disinfection with chemicals in epidemic times is, doubtless, useful in checking the development of infectious material that may chance to be delayed at any part in the course of the sewer.

With regard to gaseous disinfection, the form of proceeding which is most generally convenient for the disinfection of room-spaces, the results are generally unsatisfactory. We find that while sulphurous acid is unreliable, chlorine and bromine, though more effectual, can yet only be relied upon to destroy fully-exposed organisms, and that the simultaneous saturation of the air with watery vapour is necessary to secure their full efficiency.

Dependence upon these gaseous agents for the disinfection of clothes, blankets, and similar articles is obviously fraught with extreme danger; and yet in many populous districts the local authorities responsible for the

* As *e.g.* during the cholera Epidemic of 1866: *op. cit.*

public health have no more certain means of disinfecting such articles.

When we consider that the desquamative periods of many infectious diseases, notably smallpox and scarlet fever, are periods of extreme danger, during which infective material is given off with the dried epithelial scales in a readily portable form, and during which bedding and clothes become loaded with such infectious particles; and when we further remember that this desquamation of infectious particles takes place similarly in the mildest as in the most severe cases, it becomes at once obvious that efficient means for disinfection of such articles by hot air or steam is a first necessity, and that where such efficient means are not available, contagion is likely to be largely carried by means of "fomites"—a fact that especially applies in cases where one of these diseases has been nursed for any length of time at home.

When the articles of clothing and bedding have been removed for proper treatment, there yet remains the empty room-space to consider; and we still have to secure the destruction of the disease-germs contained in the dust which adheres to the wall-surfaces, or lurks in the chinks and crannies. Theoretically, sulphur is ineffectual as a true disinfectant; practically, we have reverted to its use, after chlorine vapour had been tried for some time; and our success is uniform. Chlorine was discarded owing to its very destructive action, which caused numerous complaints.

The non-return of a disease in a certain instance, after the use of a particular process, is, as Koch points out, no proof of the destruction of the disease-germ; but the non-return of disease in infected houses after many years' use of a process, in cases where the disease will return in the absence of disinfection, is at least some argument towards its practical utility.

It is of course possible, or it may even be said probable, that the less resistant nature of the germs of the commoner infectious diseases may explain in great part our success; but, in addition to this, the method in which disinfection is carried out has no doubt a large influence. In the first place, the window-cracks, the chimney, and the door on the outside are all carefully pasted up, so as to render the room as air-tight as possible, the proportion of sulphur burnt is large, and the operation is continued for some time. Secondly, a thorough scrubbing and free ventilation are secured: the obnoxious sulphur-smell no doubt conduces to the free securing of ventilation. And thirdly, if the patient has been nursed at home, we secure, if possible, the re-papering, liming, and thorough cleansing, in addition to fumigation, of the infected rooms. Where the patient is removed in quite an early stage, fumigation might possibly be omitted, were it not that its moral influence, as an occult process, produces good effect in impressing the nurse or friends of the patient, and in securing more ready and thorough co-operation than would be given to the ordinary and every-day process of cleansing. With these precautions, sulphur fumigation appears to me to be the least objectionable, and a fairly efficient, agent in dealing with infected spaces.

In those diseases in which desquamation of infectious particles is a marked feature, inunction with vaseline or oil, so that the body-surface is constantly moist, must prove most valuable. The action appears to be chiefly mechanical, but is most important; and repeated and thorough inunction will prevent much of the contamination of the air by infectious particles. There can of course be no objection to the use of camphor, carbolic acid, or thymol, in conjunction with the oil, and it is probably more grateful to the patient.

Similarly in such diseases, if isolated at home, the use of the wet sheet saturated and kept moist with carbolic acid solution, preferably mixed with glycerine, is useful in many ways. I believe its use is chiefly mechanical, in arresting infectious particles; but it also serves as an ever-present warning of danger, and of the necessity for continued precaution.

It is hardly necessary to point out that certain popular devices, such as the placing of Condy's fluid, or of carbolic powder, about the room in saucers, can be of no practical use so far as any hoped-for disinfectant action is concerned. As deodorants they may to some extent suffice; but fresh air, and plenty of it, is in every case of infectious disease imperative, and the use of any chemical deodorant must, in all cases, rank as of secondary importance.

### THE LEGAL POSITION OF A SANITARY AUTHORITY WITH RESPECT TO THE QUESTION OF DISINFECTION.*

It is the duty of any Sanitary Authority, when they are of opinion that the cleansing and disinfecting of any house or part thereof, and of any articles therein likely to retain infection, are necessary, to serve a notice upon the owner or occupier, requiring him to cleanse and disinfect such house or part thereof, and articles, within a time specified in the notice; and should the owner or occupier make default, he is liable to a continuing penalty of 10s. daily, and the local authority may thereupon cause the house

* For the law upon this subject, consult Dr. Hime's excellent *Practical Guide to the Public Health Act*, 1875. Baillière, Tindall, and Cox. London. 1884.

and articles to be cleansed and disinfected, and may recover the expenses summarily. Provided also that where the owner or occupier is from poverty or otherwise unable to effectually carry out these requirements, the authority may themselves cleanse and disinfect the house and articles, and defray the expenses thereof. (*Public Health Act*, 1875. Sec. 120.)

Any Sanitary Authority may direct the destruction of infected bedding, clothing, or other articles, and may give compensation for the same. (Sec. 121.)

Any Sanitary Authority may provide a proper place, with all necessary apparatus and attendance, for the disinfection of bedding, clothing, or other infected articles and may cause any articles brought for disinfection to be disinfected free of charge. (Sec. 122.)

Any person who lends, sells, transmits, or exposes, without previous disinfection, any infected bedding, clothing, rags, or other things, is liable to a penalty of £5; and any person who knowingly lets for hire, without proper previous disinfection, any house, room, or part of a house which has become infected, is liable to a penalty of £20, and to a similar penalty or to imprisonment for one month for making false statements respecting the existence of infectious disease in such house. And it is the duty of the Sanitary Authority to secure the enforcement of these penalties.

Such are the statutory duties and powers of a Sanitary Authority with regard to disinfection; but as the position of an authority in its relation to the public is untenable unless its powers are helpfully enforced, and as it is obvious that householders can have neither the knowledge nor the appliances necessary to efficiently carry out processes of disinfection themselves, it is very advisable that the

Sanitary Authority should undertake the carrying out of all such necessary disinfection upon request.

For this purpose, the Bristol Authority keep a staff of men for disinfecting purposes, who will, upon due notice, be sent, under the District Inspector's supervision, to fumigate and direct the subsequent cleansing of rooms on behalf of the owner or occupier. Similarly, the Authority have, for the convenience of the public, undertaken to send for clothes and bedding in a properly covered cart, by which the infected articles are, during transmission, secured against exposure; to disinfect them thoroughly in the steam apparatus, but not to wash them; and to return them safely. A small charge is made by the Authority for carrying out these processes of disinfection, where the applicants are in a position to pay; *but in no instance is the question of payment allowed to interfere with or to delay the necessary work.*

In conclusion, it would appear that while heat-methods have alone stood the test of accurate experiment, the other accepted methods of disinfection have been shown to be of variable and, with few exceptions, of very limited efficacy. These methods, however, when carried out with completeness and intelligence, and with due recognition of their limited power,—but only under these conditions,—are still reliable weapons in our hands; so that while, on the one hand, it behoves the authorities charged with the maintenance of the public health to provide a sufficient trained staff and the necessary appliances for carrying out processes of disinfection; it is, on the other hand, obviously to the interest of householders to entrust all necessary disinfection to their hands, so that no additional danger may be incurred through its imperfect or incomplete performance.

## Figures and Tables

**Figure f1:**